# Simulation of Somatic Evolution Through the Introduction of Random Mutation to the Rules of Conway’s Game of Life

**DOI:** 10.1007/s12195-024-00828-9

**Published:** 2024-10-20

**Authors:** Michael R. King

**Affiliations:** Department of Bioengineering, Rice University, Houston, TX USA

**Keywords:** Cellular automata, Game of life, Somatic evolution

## Abstract

**Introduction:**

Conway’s Game of Life (GOL), and related cellular automata (CA) models, have served as interesting simulations of complex behaviors resulting from simple rules of interactions between neighboring cells, that sometime resemble the growth and reproduction of living things. Thus, CA has been applied towards understanding the interaction and reproduction of single-cell organisms, and the growth of larger, disorganized tissues such as tumors. Surprisingly, however, there have been few attempts to adapt simple CA models to recreate the evolution of either new species, or subclones within a multicellular, tumor-like tissue.

**Methods:**

In this article, I present a modified form of the classic Conway’s GOL simulation, in which the three integer thresholds that define GOL (number of neighboring cells, below which a cell will “die of loneliness”; number of neighboring cells, above which a cell will die of overcrowding; and number of neighboring cells that will result in spontaneous birth of a new cell within an empty lattice location) are occasionally altered with a randomized mutation of fractional magnitude during new “cell birth” events. Newly born cells “inherit” the current mutation state of a neighboring parent cell, and over the course of 10,000 generations these mutations tend to accumulate until they impact the behaviors of individual cells, causing them to transition from the sparse, small patterns of live cells characteristic of GOL into a more dense, unregulated growth resembling a connected tumor tissue.

**Results:**

The mutation rate and mutation magnitude were systematically varied in repeated randomized simulation runs, and it was determined that the most important mutated rule for the transition to unregulated, tumor-like growth was the overcrowding threshold, with the spontaneous birth and loneliness thresholds being of secondary importance. Spatial maps of the different “subclones” of cells that spontaneously develop during a typical simulation trial reveal that cells with greater fitness will overgrow the lattice and proliferate while the less fit, “wildtype” GOL cells die out and are replaced with mutant cells.

**Conclusions:**

This simple modeling approach can be easily modified to add complexity and more realistic biological details, and may yield new understanding of cancer and somatic evolution.

**Supplementary Information:**

The online version contains supplementary material available at 10.1007/s12195-024-00828-9.

## Introduction

Conway’s Game of Life (GOL), one of the simplest and best known forms of cellular automata (CA) models, has fascinated both mathematicians and lay people alike since its introduction in 1970 [4]. Starting with an initial state of “dead” or “live” cells on a square lattice, some cells die while others are born, based on the current state of each lattice point’s 8 adjacent neighbors and a limited set of rules. The rich and surprising behaviors that emerge, with collections of cells growing in size, decaying to a dead state, migrating across the lattice, or even reproducing exact copies of themselves, have suggested to many a representation of virtual “life” encoded within. Perhaps the most extensive characterization of the range of behaviors in CA that arise in response to various initial conditions and rule sets was carried out by Stephen Wolfram, in his 1200-page book A New Kind of Science [24], in which it was proposed that such complexity arising from apparent randomness might explain natural phenomena such as complexity in biology, fundamental theories of physics, and even the very nature of mathematics and intelligence.

The essential elements of evolution are twofold: (i) random variation, and (ii) natural selection. These elements are present in the generation of new species, as described by Darwin [3], as well as in somatic evolution, i.e., the mutation and fitness competition of cells within a solid tissue such as a tumor, occurring within the lifespan of an individual organism [15]. So, is GOL more akin to a simulation of Darwinian, or somatic evolution? One of the central features of GOL is that the 2-D spatial organization of surviving cells is preserved throughout the simulation on a square lattice. However, this feature would be less relevant for Darwinian evolution, except for certain immobile species or in the most coarse-grained sense, such as the patterns of speciation in different regions of the U.S., or on the individual land masses of the Galápagos Islands for instance. Somatic evolution in solid tumors, on the other hand, is well documented to exhibit spatial mosaics of individual subclones of cells, with microdissection used in combination with single cell genomics enabling the reconstruction of ancestral relationships in space and time [9]. In addition, while Darwinian evolution most commonly involves the combining of genetic code from two distinct parents, somatic evolution involves a form of asexual reproduction from a single parent cell. Noting that classic GOL rules dictate a new daughter cell arises when 2 or 3 parent cells are adjacent, and allowing for this spawning rule to be mutated further to include 1–4 + nearby parents, the most robust and versatile form of GOL that passes traits to offspring would be to dictate a single parent, randomly selected from eligible neighbors when prompted. For all of these reasons, I have chosen to modify GOL by introducing random mutation into the rule thresholds with the expectation that it may serve as a useful model of the somatic evolution of cells within a growing mass.

There have been many attempts to model the growth of solid tumors using so-called “hybrid” cellular automata models in 2D or 3D [1, 2, 5–8]; [10–14, 18, 20–23, 25]. While part of the appeal of pure CA models is that complex and collective patterns of behavior of cells on a spatial lattice can arise from a small number of simple rules governing the interactions between neighboring cells, subject to different initial conditions, these cancer models depart from that strict formalism by adding complexities that represent more biological realism. For instance, cancer CA models will often superimpose the calculation of spatial gradients of diffusible species such as oxygen, glucose, chemoattractants, cytokines, and angiogenic factors. Some other common cancer modifications which make tumor models “hybrid” in nature rather than pure CA include: cell migration; multiple interacting cell types and proliferation states; predetermined cell lifespans, where cells expire upon reaching a certain age; growth of blood vessels which feed the tumor periphery with nutrients; and a buildup of internal pressure which affects proliferation and molecular transport. Such hybrid CA tumor growth models have produced virtual tumors which grow with a circular/spherical shape and can exhibit a somewhat rough, random periphery that resembles real tumors. Some of these models recreate growth to a macroscopic ~ 1 mm diameter and then predict a necrotic core before vascularization occurs, another feature of real experimental or clinical tumors. Some models have shown conditions where interactions with immune cells can suppress growth, while other models have explored in the hybrid CA setting how drug treatment affects the dynamics of growth as well.

While a vast range of CA rule sets, initial conditions, multiple cell states beyond live/dead, and different lattice geometries have been explored (see, for instance, [24]), to my knowledge, no one has ever explored a version of GOL in which the static thresholds defining the GOL rules for birth, survival and death are allowed to randomly mutate and then cells with incrementally varying individual (and heritable) rules compete to out-proliferate each other. One outlier is an open source program called “SproutLife”, developed by Alex Shapiro and disseminated on GitHub (https://github.com/ShprAlex/SproutLife) and YouTube (https://www.youtube.com/@shpralex). SproutLife was designed to simulate the growth and evolution of small, multicellular “organisms” with an “open-ended genome” where individual collections of cells can randomly incorporate new phenotypes (such as accelerated growth, migration, the ability to “battle” other organisms that each collection of cells encounters) from a limited, prescribed list. While interesting as a self-contained simulation of competing organisms, SproutLife represents a major departure from the original GOL and thus is not directly comparable to the present study.

In the following sections, I will describe the modifications made to an existing GOL simulation implemented in Matlab. Randomized mutations in the numerical thresholds define the simple rule set of the resulting CA model, in which newly born cells inherit the mutated rule set from a neighboring parent cell. I then present a summary of representative results obtained for a randomized initial lattice configuration and a wide range of mutation rates and mutation magnitudes, and draw some general conclusions based on these observations. Finally, I comment on the possible implications of this work as it relates to the biological phenomenon of somatic evolution, and suggest some interesting future directions for simulations of evolutionary processes based on this framework.

## Methods

Since the conventional GOL algorithm has been so well documented and explored for nearly a half century, I will focus the description here on the modifications necessary to adapt a freely available GOL program implemented in Matlab to introduce random mutation into the GOL rules. I took as my starting point the Conway’s Game of Life program developed by Dimitrios Piretzidis [17] and distributed at the MathWorks.com website. The program features a GUI interface and can be executed with a randomized live/dead initial 2-D square lattice condition of specified size or can take a predefined .mat file as input to define a prescribed initial condition. The downloadable zip file includes some classic GOL initial configurations as .mat files, such as the glider, glider gun, and two symmetrical cross configurations. All of the simulation results presented in this article were run with the default lattice size of 100x100, a maximum of 10,000 generations before termination, and with a randomly generated initial population that assigns 50% of the lattice nodes with live cells.

The Piretzidis Matlab program is a standard Conway’s GOL simulation, with an 8-neighbor Moore neighborhood on a 2-D square lattice, and the following rule set:

- Any live cell with fewer than two live neighbors dies, the so-called “loneliness” rule.
- Any live cell with two or three live neighbors survives to the next generation. This rule is equivalent to: “above the threshold for loneliness and below the threshold for overcrowding = survival” and thus does not represent its own distinct numerical threshold subject to mutation per se.
- Any live cell with more than three live neighbors dies, the so called “overcrowding” rule.
- Any dead cell with exactly three live neighbors becomes a newly born live cell.

The program avoids the need for a neighborhood boundary condition, by not testing the edge nodes for cell birth and death. The original program, when executed, replots the lattice at each generation, displaying the current cell population and generation number, and terminates when the population reaches a steady-state, or reaches the maximum generation number, whichever occurs first, and then plots the population as a function of generation number at the conclusion of each simulation.

The modified program, including random mutation events to either the (i) lonely, (ii) born, or (iii) crowded thresholds during a fraction of new cell birth events, is included as Supplemental File 1. First, to track mutations in these thresholds and allow parent cells to pass on their current mutation state to progeny cells, three new 2-D arrays of equal dimension to the original lattice were created: lonely, born and crowded, with initial threshold values set to 2/3/3 respectively in accordance with the original Conway GOL rules. The mutation rate is defined as the average fraction of new cellular births which result in a mutation, ranging from 0–1 and compared against a randomly generated number uniformly distributed between 0 and 1. The mutation magnitude is a scalar factor which multiplies a normally distributed random number with mean = 0 and standard deviation = 1, which is then added to one of the three threshold values, after random selection during births that are determined to be a successful mutation event. Whereas the original program extracts the Moore’s neighborhood of each cell (“small_universe”) from the full lattice (“universe”) and passes it to a subroutine that enforces the GOL rules (“f_rules”), the modified program must also determine the three threshold values to pass those values (“tiny_lonely”, “tiny_born”, “tiny_crowded”) to the f_rules subroutine, after rounding each (potentially) mutated value to the nearest integer prior to rules enforcement. If a new cell is born and a mutation event determined to occur, one of the live neighbors in the Moore neighborhood is randomly selected and its current mutation state passed on to the newly born cell, together with any new mutation which may have occurred at the current iteration. When a cell dies, it’s lattice point is reassigned the original GOL threshold values, for the purposes of tracking the mutation state of the whole lattice. Finally, at the conclusion of each simulation run, a 2-D map of the final mutation state of the three thresholds at each lattice point was plotted for inspection. 58 simulation runs were carried out while varying the mutation rate from 0, 0.01, 0.05, 0.1, 0.2, 0.4, 1.0, and varying the mutation magnitude from 0.25, 0.5, 0.75, 1.0, 1.5, 2, 3, 4, 5, 6 and 10.

## Results

**Fig. 1:**
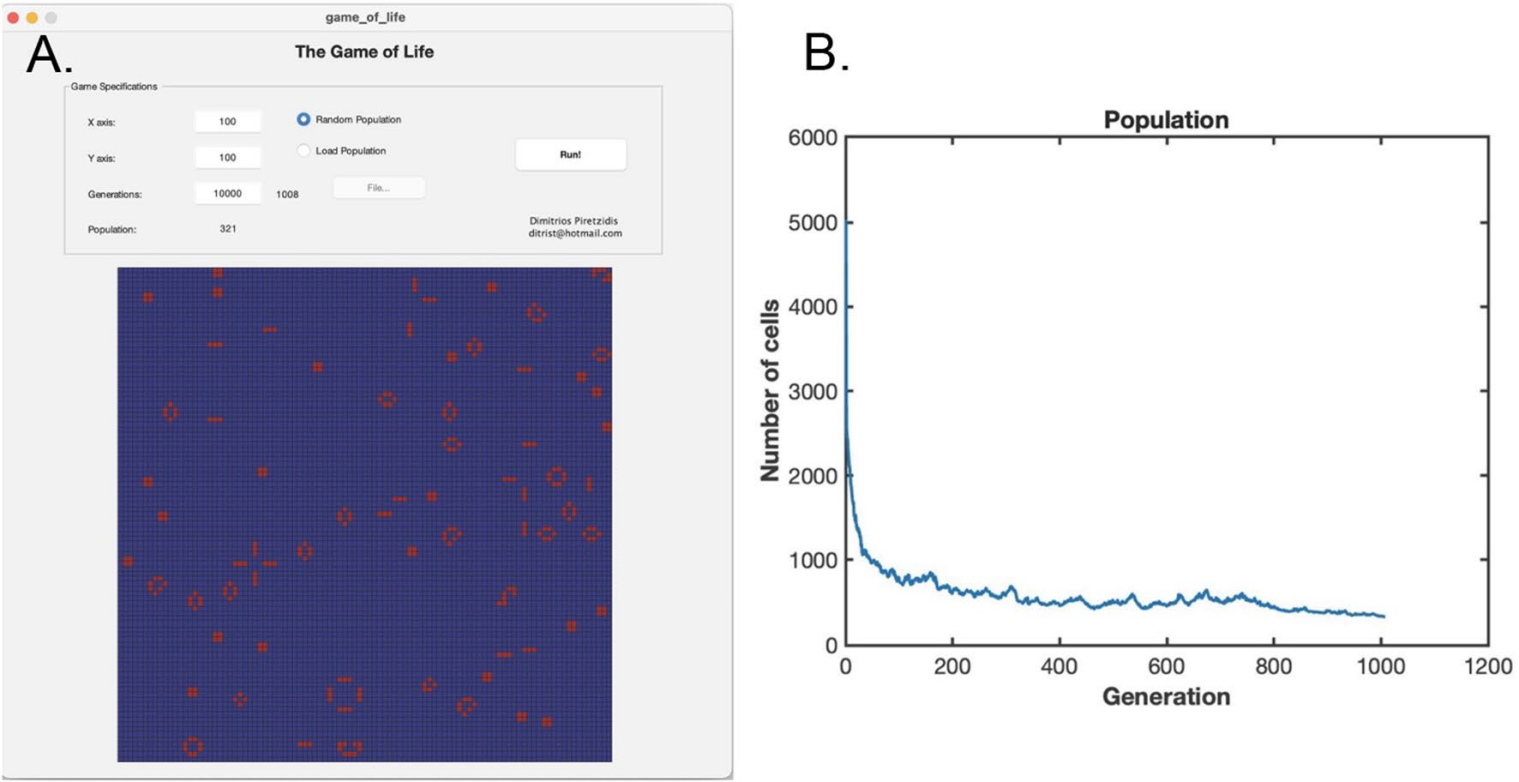
Dynamics of Conway’s Game of Life with no mutation. **A** Final population on the 2-D lattice, showing live cells in red and dead/empty cells in blue. **B** Cell population as a function of generation number

Figure 1 shows the dynamics of a typical GOL simulation in the absence of rule mutation. The initial population of 5000 cells (50% random lattice occupancy, by default) shows a rapid decrease of early generations, followed by a slower, noisy and approximately linear decrease in population until steady-state is reached. After about 1000 generations the lattice reaches a steady-state where remaining live cells are either stationary or continue to undergo small repeating fluctuations, at which point the simulation terminates. Supplementary Video 1 shows an animation of a representative simulation run in the absence of mutation (mutation rate = 0).

Numerical results from the GOL simulations with nonzero mutation rate are presented in Supplementary File 2, consisting of the “end generation” (final number of generations executed, until either reaching steady-state or the maximum generation number of 10,000), “end population”, the final number of live cells at the conclusion of the simulation, and the final mean value of the lonely, born, and crowded thresholds averaged across the 2-D lattice, as a function of mutation rate and mutation magnitude. These results are graphically presented in the next five figures, with animations of representative simulations shown in several supplemental videos.

**Fig. 2:**
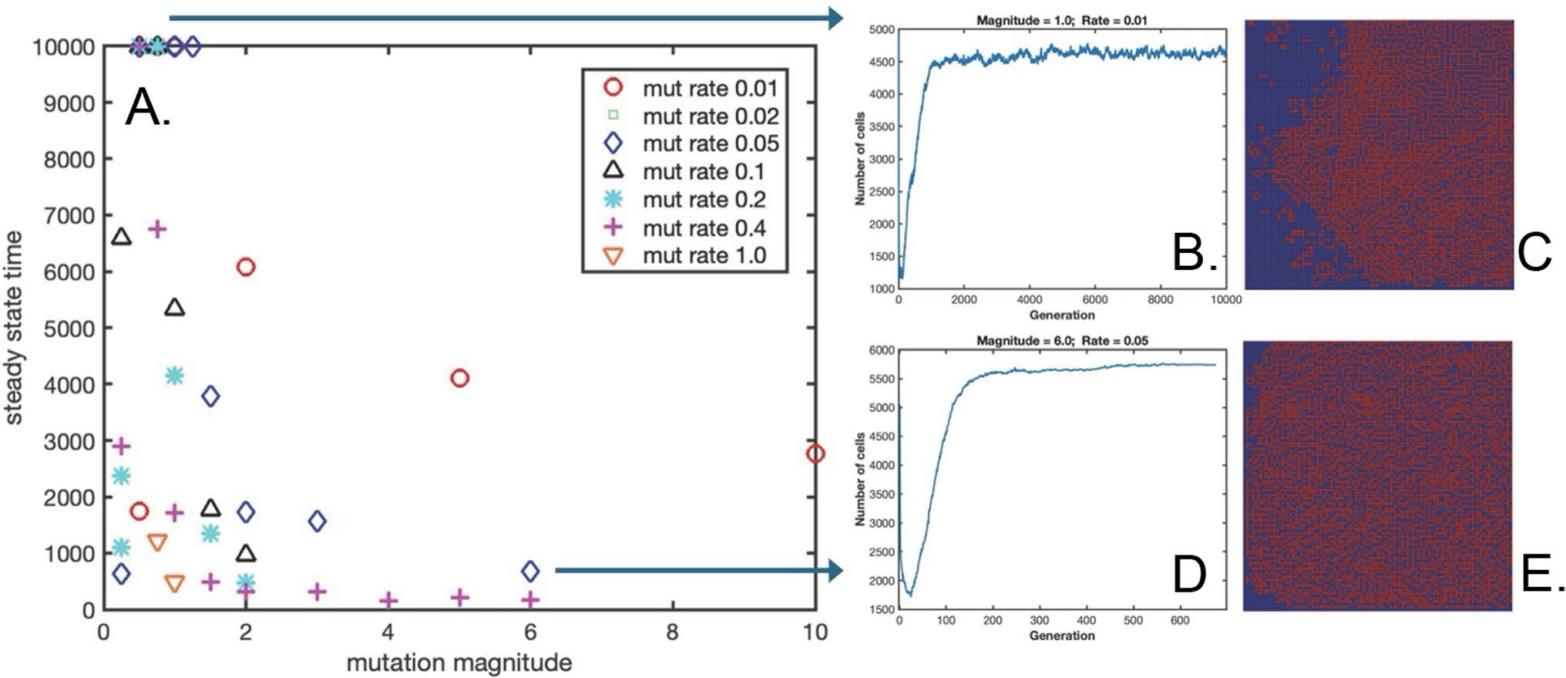
**A** Final number of generations before reaching steady-state or maximum generation number, as a function of mutation magnitude and mutation rate. **B** Population of live cells as a function of generation number, for mutation rate = 0.01 and mutation magnitude = 1.0. C. Final lattice map of cell population, with live cells in red and dead/empty cells in blue. D. Population of live cells as a function of generation number, for mutation rate = 0.05 and mutation magnitude of 6.0. E. Final lattice map of cell population, with live cells in red and dead/empty cells in blue

Figure 2A shows the number of generations necessary to reach steady-state (or the maximum of 10,000 generations in the case of no steady-state reached) as a function of mutation magnitude and mutation rate. Figure 2B–E show the cell population vs. generation number, and a final spatial map of live cells for a mutation rate of 0.01 and mutation magnitude of 1.0, and for a mutation rate of 0.05 and mutation magnitude of 6.0, respectively, with animations of these two conditions presented in Supplementary Videos 2 and 3. In general, as mutations are gradually introduced at the lowest magnitudes studied, the number of generations until steady-state is reached increases, reaching a maximum of 10,000 generations (i.e., no steady-state reached), and then decreasing back down below 1000 total generations at the highest mutation magnitudes. Another general trend observed is that the total number of generations to reach steady-state decreases with increasing mutation rate, as expected. When examining the simulations with significant mutation, the most striking observation is the formation of a densely packed area of surviving cells, much like the uncontrolled growth of a tumor (Fig. 2C, E; Supplementary Videos 2 and 3), rather than the sparse population of < 5% final live cell occupancy seen in the original GOL (Fig. 1). For a mutation magnitude of 1.0, it is observed that rather than overgrow the entire lattice, two diagonal boundaries between live and dead cell regions persisted, with considerable cell fluctuations which prevent the simulation from reaching a terminal steady-state (Fig. 2B, C; Supplementary Video 2). However, at the higher mutation magnitude of 6.0, the uncontrolled “tumor-like” growth rapidly takes over the entire lattice in under 700 generations, thus terminating the simulation once a static equilibrium is reached.

**Fig. 3:**
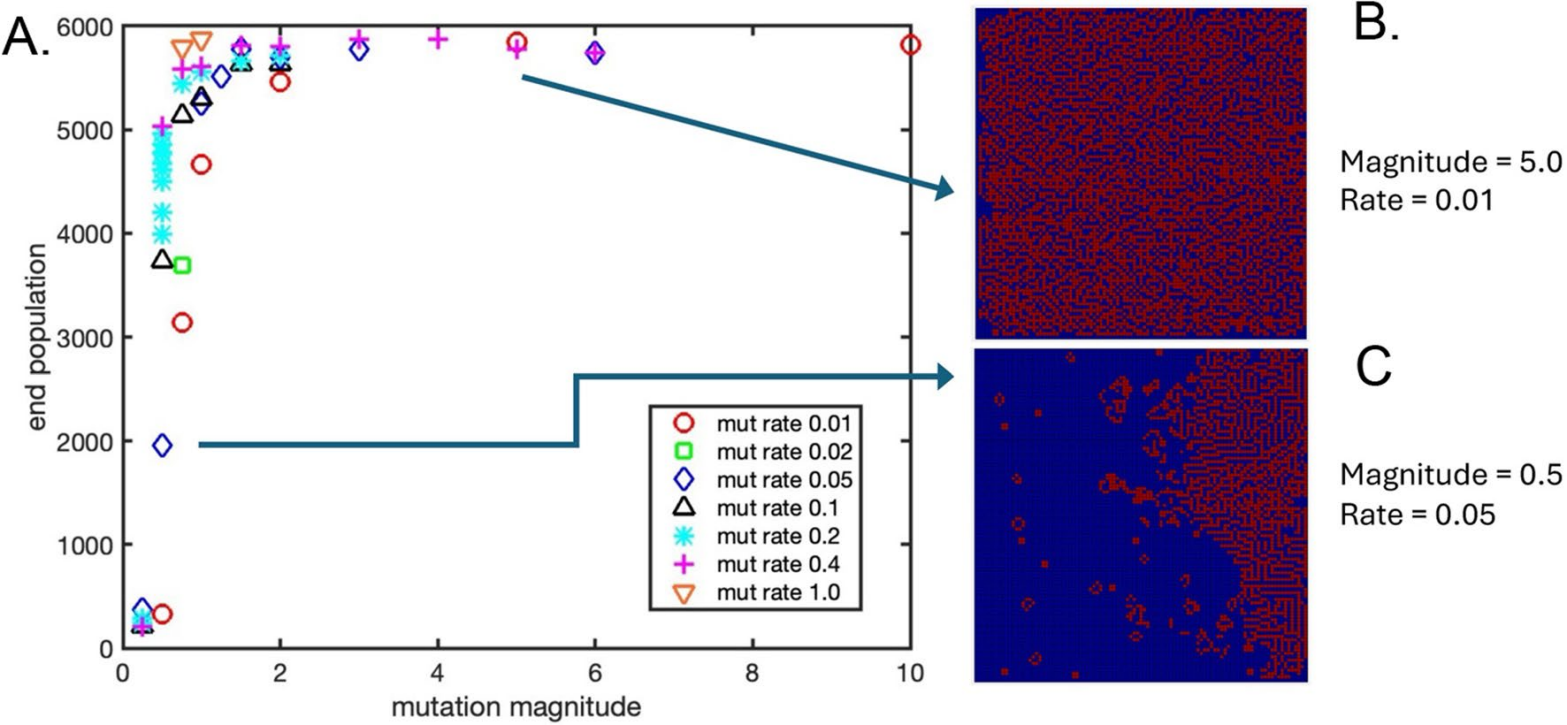
**A** The final live cell population at the end of each simulation, as a function of mutation magnitude and mutation rate. **B** Final spatial map of live cell population for a mutation magnitude = 5.0 and mutation rate = 0.01. **C** Final spatial map of live cell population for a mutation magnitude = 0.5 and mutation rate = 0.05

Figure 3A shows the final live cell population size as a function of mutation magnitude and mutation rate. Final population was found to be a strong function of mutation magnitude over a transitional region of 0–2, and then relatively independent of mutation magnitude at higher values, while a weaker function of mutation rate. It appears that a critical mutation magnitude (the parameter that multiplies a randomly generated Gaussian mutation of mean = 0 and standard deviation = 1.0) exists around 0.5, above which a transition to uncontrolled, dense tumor-like growth is first observed. Note that due to the rounding step before GOL rule thresholds are passed to the rule enforcement subroutine, the total accumulated mutation of any of the three rule thresholds (lonely, born, crowded) must reach at least 0.50 in magnitude in either direction to register any effect on the GOL algorithm, although smaller, fractional mutations are still tracked and can grow or shrink over time with new mutation events. The final live cell maps for two conditions, mutation magnitude = 5.0 and mutation rate = 0.01, and mutation magnitude = 0.5 and mutation rate = 0.05, are presented in Fig. 3B, C with accompanying animations in Supplementary Videos 4 and 5, respectively. At the higher mutation magnitudes, a dense, tumor-like growth rapidly overtakes the entire lattice, whereas at the critical mutation magnitude of 0.5 we observe a tumor-like growth on one side, which reaches a steady-state in apparent equilibrium with a sparse, Conway GOL-like region of mostly dead cells, with significant fluctuation at the boundary involving “glider”-like projections in and out of the boundary between the two regions.

**Fig. 4:**
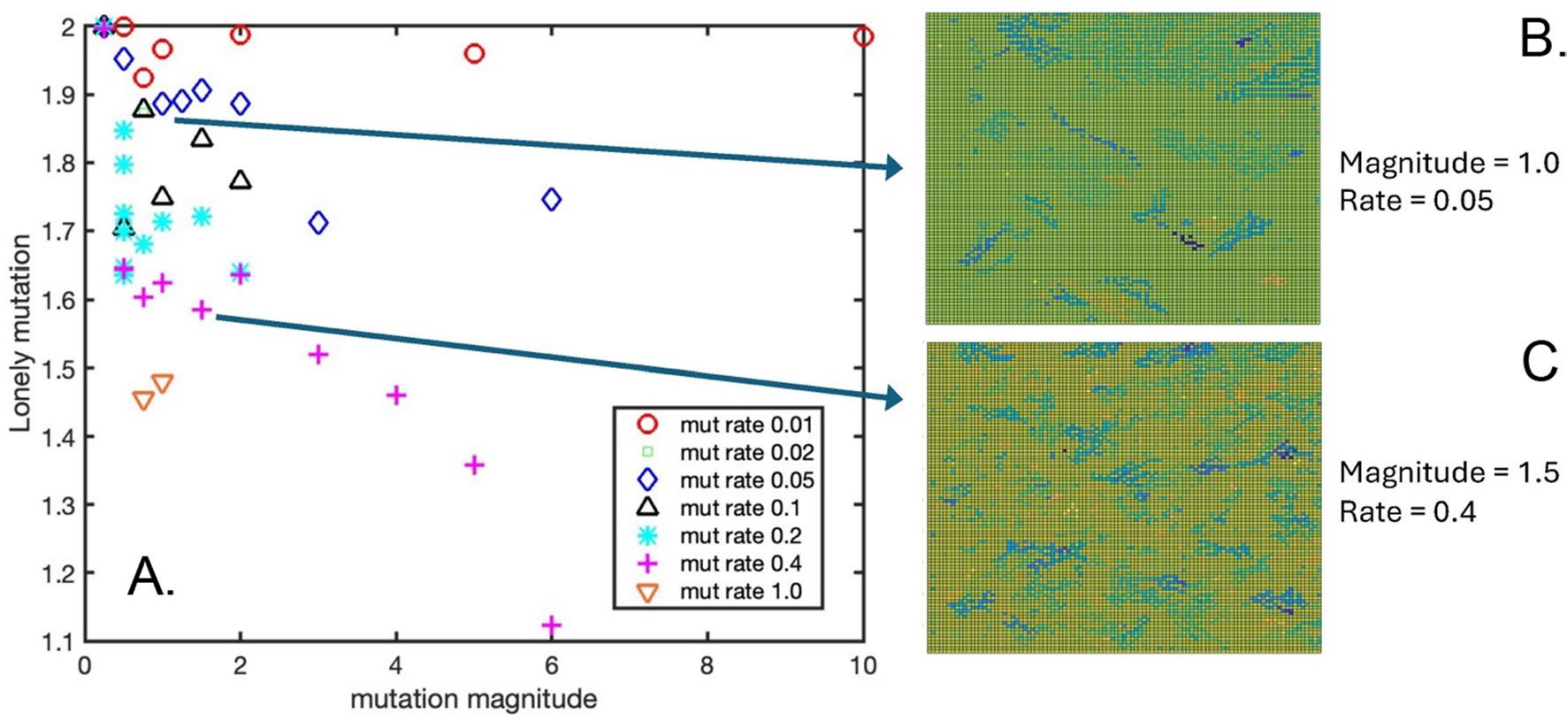
**A** The final lonely GOL threshold value averaged over the 2-D lattice, as a function of mutation magnitude and mutation rate. **B** A spatial map of the final lonely GOL threshold for a mutation magnitude = 1.0 and mutation rate = 0.05. **C** A spatial map of the final lonely GOL threshold for a mutation magnitude = 1.5 and mutation rate = 0.4

Figure 4A shows the final mutated value of the “lonely” GOL threshold (i.e., the minimum number of live Moore neighbors, below which a live cell will die of loneliness), averaged across the 2-D square lattice and plotted as a function of mutation magnitude and mutation rate. Note that the starting value of this threshold is 2.0 in accordance with Conway’s GOL. Interestingly, during the course of each simulation the average lonely threshold is mutated only in the negative direction, with final values ranging between 1.1 and 2.0. In general, the lonely threshold shows greater extent of mutation for increasing mutation rates and increasing mutation magnitudes. Fig. 4B, C shows the final spatial color maps of the mutated lonely threshold for two specific simulation conditions: mutation magnitude = 1.0 and mutation rate = 0.05, and mutation magnitude = 1.5 and mutation rate = 0.4, respectively. Animations of the modified GOL simulation for these two cases are presented in Supplementary Videos 6 and 7. While the light green regions in Fig. 4B, C indicate lattice points with a final lonely threshold close to the initial value of 2.0, blue, yellow, and dark green patterns show clusters of mutated cells which have conferred their GOL rule from parent cell to daughter cell, and thus represent a record of the spatial patterns that subclones followed during their propagation across the lattice.

**Fig. 5:**
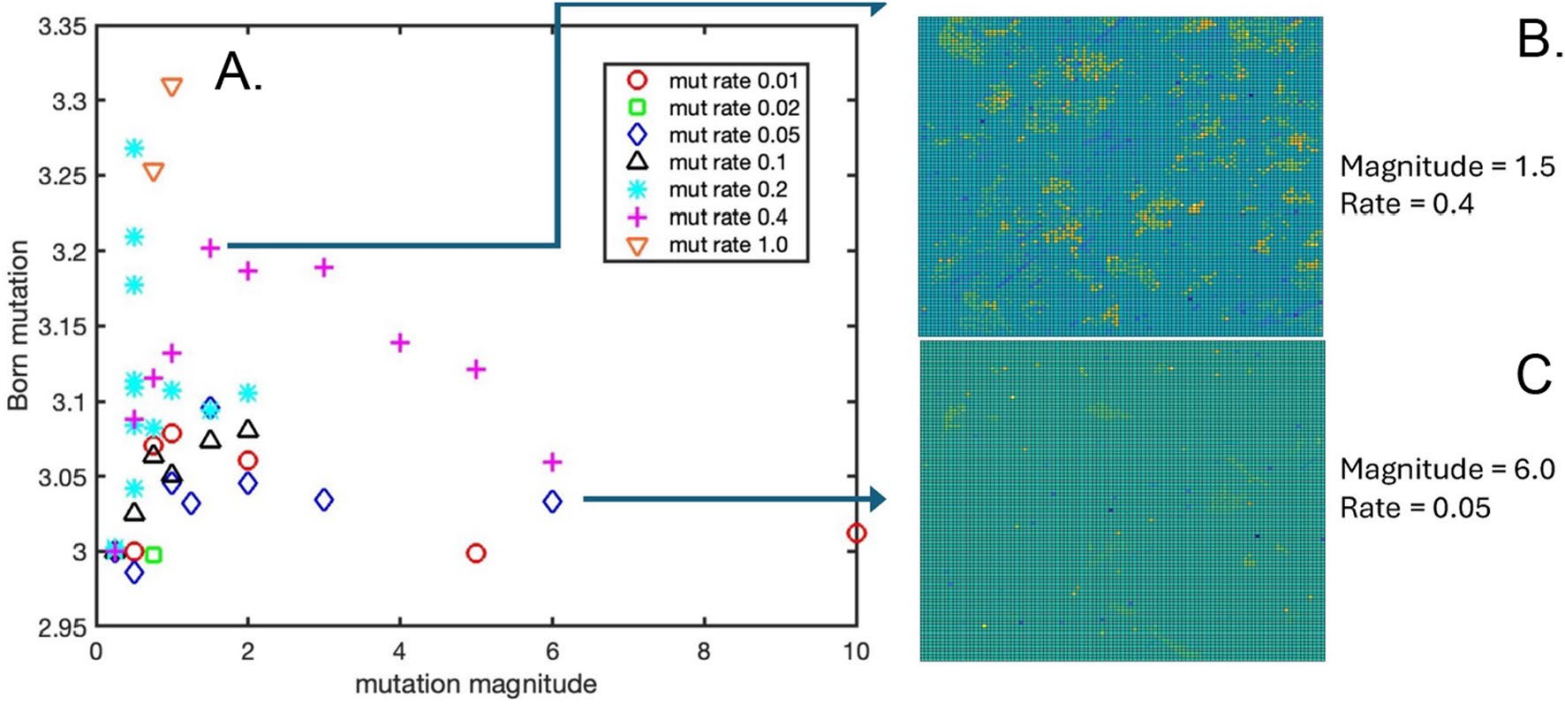
**A** The final born GOL threshold value averaged over the 2-D lattice, as a function of mutation magnitude and mutation rate. **B** A spatial map of the final born GOL threshold for a mutation magnitude = 1.5 and mutation rate = 0.4. **C** A spatial map of the final born GOL threshold for a mutation magnitude = 1.5 and mutation rate = 0.4

Figure 5A shows the final mutated value of the “born” GOL threshold (i.e., when rounded to an integer value, the number of live Moore neighbors for which a spontaneous cell birth will occur in an empty center lattice point), averaged across the 2-D square lattice and plotted as a function of mutation magnitude and mutation rate. Note that this average mutated threshold does not stray far from the initial value of 3.0, ranging from 2.98–3.31. This suggests that born threshold mutations which round to either 2 or 4 may not promote enhanced cell proliferation (and expansion of a new mutant), thus larger mutations may represent a “loss of function” with reduced cell fitness. In general, the magnitude of final mutation is an increasing function of mutation rate, whereas the average mutation seems to exhibit a bimodal dependence on mutation magnitude, increasing with increasing mutation magnitudes between 0 and 1, reaching a plateau around 1–2, and then showing an inverse/decreasing dependence on mutation magnitudes > 2. Fig. 5B, C shows final spatial color maps of the mutated born threshold for two specific simulation conditions: mutation magnitude = 1.5 and mutation rate = 0.4, and mutation magnitude = 6.0 and mutation rate = 0.05, respectively. Animations of the modified GOL simulation for these two cases are presented in Supplementary Videos 7 and 3, respectively. Interestingly, the simulation corresponding to Fig. 5C and Supplementary Video 3 rapidly reached saturation of the lattice in only 676 generations (see Supplementary Table 1) due to the very high mutation magnitude, leaving little time for mutations of this threshold to accumulate (Fig. 5C).

**Fig. 6:**
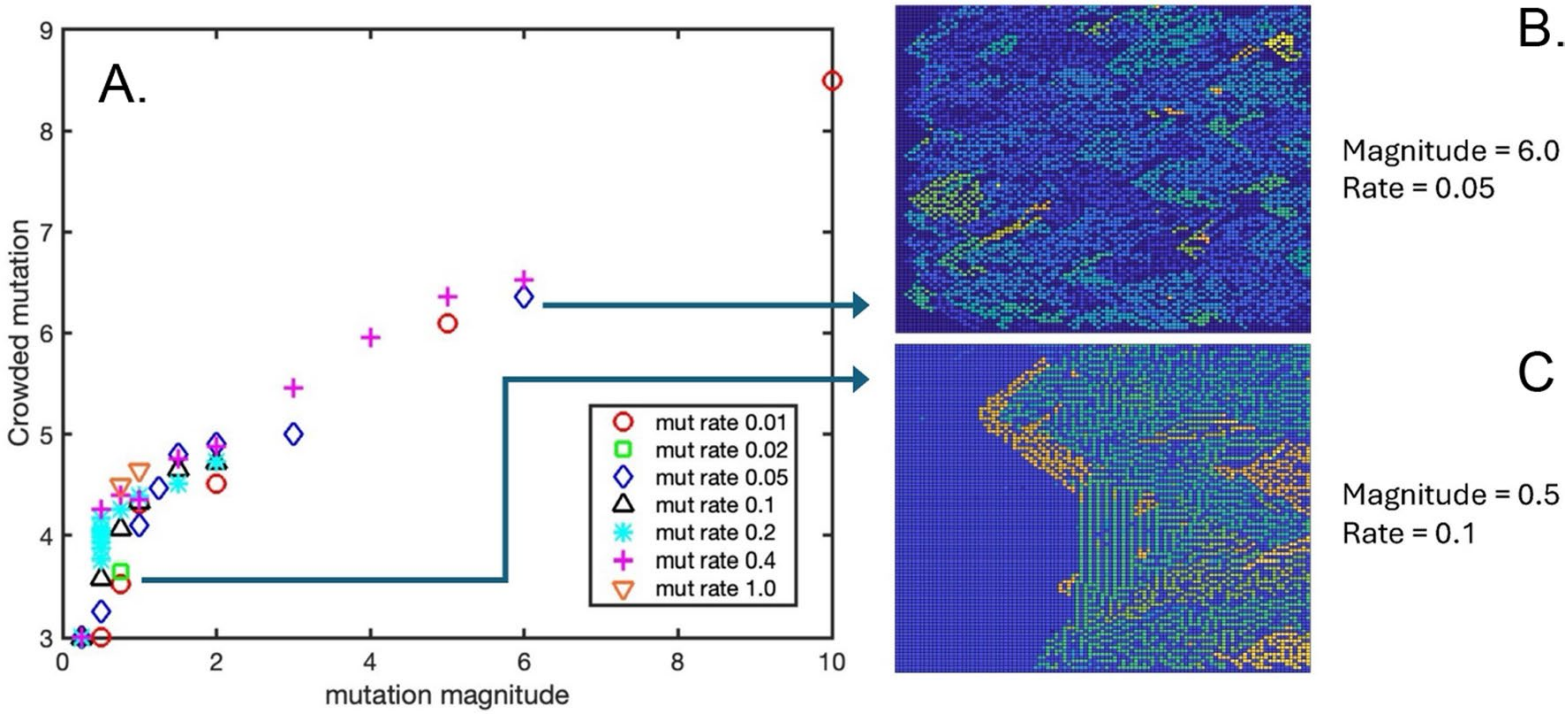
**A** The final crowded GOL threshold value averaged over the 2-D lattice, as a function of mutation magnitude and mutation rate. **B** A spatial map of the final crowded GOL threshold for a mutation magnitude = 6.0 and mutation rate = 0.05. **C** A spatial map of the final crowded GOL threshold for a mutation magnitude = 0.5 and mutation rate = 0.1

Figure 6A shows the final mutated value of the “crowded” GOL threshold (i.e., the maximum number of live Moore neighbors, above which a live cell will die of overcrowding), averaged across the 2-D square lattice and plotted as a function of mutation magnitude and mutation rate. One striking difference in this GOL threshold, compared to the lonely and born thresholds, is the much greater magnitude of the average final mutations, which range from the initial value of 3.0 up to 8.5. For comparison, the average final lonely threshold ranged from 2.0 down to 1.1 (Fig. 4) and the average final born threshold ranged from 2.98 (slightly lower than initial) up to 3.31. This greatly increased range in the mutated values of the crowded threshold strongly suggests that elevating this GOL rule above 3.0 likely confers an “evolutionary” advantage and increased cellular fitness thus promoting survival, much more so than the other two rules. Remarkably, the final average value of crowded threshold appears to be independent of mutation rate, with the various values plotted in Fig. 6A collapsing onto a single curve that increases with increasing mutation magnitude, something not observed in the other two GOL rules (Figs. 4A, 5A). Figure 6B, C show the final 2-D spatial maps of the crowded threshold at each lattice point for two different simulation conditions: mutation magnitude = 6.0 and mutational rate = 0.05, and mutation magnitude = 0.5 and mutation rate = 0.1, respectively. These maps are also strikingly different in appearance to the maps of the lonely and born thresholds (Figs. 4B, C and 5B, C) in that they show much more structure and widespread regions of distinct “subclones” of mutants. Animations of the simulations corresponding to the conditions highlighted in Fig. 6B, C can be found in Supplementary Videos 3 and 8, respectively.

## Discussion

This study was initiated by first posing the question: if rare, randomized and inheritable mutation was introduced into the basic rules of the classic cellular automata model known as Conway’s Game of Life, would a modified rule set with increased fitness for proliferation and survival emerge, and gradually overtake the “wildtype” cells of the lattice? To address this unanswered question, I introduced such a mutation mechanism to the three distinct thresholds that define the GOL rules: the lonely threshold, the born threshold, and the crowded threshold, thereby providing one of the two essential elements for the process of evolution, random variation. The other essential element is of course natural selection, and a priori it seemed quite plausible that this essential ingredient of evolution might exist as well, with more advantageous local rule sets resulting in greater proliferation and survival and causing cells to pass on their improved traits to neighboring progeny cells in the lattice. Remarkably, the emergence of dominant mutant versions of the GOL cell did in fact emerge once the mutation magnitude parameter was raised to at least 0.5, creating a tumor-like bloom of surviving cells that spread across the lattice as a new phenotype that appears to be qualitatively different in its proliferation dynamics compared to the sparse cellular survivors of the original GOL. It is clear from the results presented here that the overcrowding rule is the dominant one in producing this transition to the emergence of mutant species, suggesting that the other two thresholds are relatively unimportant as mutating “genes” in this process.

The appearance of the final 2-D spatial maps of the crowded threshold (Fig. 6B, C) show a clear, qualitative difference from the spatial maps of the crowded (Fig. 4B, C) and born (Fig. 5B, C) thresholds. Only the final crowded threshold maps exhibit a tumor-like 2-D space that is essentially “tiled” with regions of different mutated subclones, as opposed to the lonely and born maps which only show localized linear trails or small blooms of mutation that are separated from each other by larger expanses of light green, i.e., approximately wildtype rule set. This strongly suggests that it is only the crowded rule that carries the information essential for conferring the evolutionary advantage that transitions the population into a tumor-like uncontrolled growth phenotype. Interestingly, the spatial maps of the crowded mutation share great similarity in appearance to the paradigm of somatic evolution as depicted in various artistic renditions of this biological process as it progresses in space and time, recognizable to students and practitioners of cancer biology [16, 19].

Interestingly, over a range of GOL simulations with rule mutation we saw a maximum occupancy of live cells of about 59% of the lattice points, much higher than the 2.9 ± 0.3% (st. dev.) occupancy of the standard GOL. Note that this maximum occupancy is equal to the arithmetic mean of 5/9 and 5/8 (0.5902), that is, 5 cells occupied in a Moore’s neighborhood of 8 (not counting the center lattice point) averaged with a neighborhood of 9 (counting the center point as occupied). Another notable observation is that in many of the simulations resulting in uncontrolled growth, the tumors tend to grow from right to left, which may be a consequence of the order in which the 2-D lattice is updated within nested loops and the accumulation/propagation of mutations across the lattice.

The current study is not without its limitations. The primary limitation is the vast array of potential simulations with varied parameter values that were not fully explored due to time constraints and scope of the study. While much attention was paid to the two parameters mutation rate and mutation magnitude, new features that are not present in the classical Conway GOL, some default parameters of the original GOL implementation developed for Matlab by Piretzidis [17] were not varied. For instance, the 100x100 lattice size was not varied, as it was assumed that this is large enough that edge effects will play a minimal role in the overall dynamics. Likewise, the randomized 50% occupancy of the initial condition of the lattice was not varied. It is possible that selecting a nonrandomized, structured, or at least uniform initial condition might be able to differentiate more subtle differences in the effects of varying the mutation parameters, without necessitating repeated simulation runs to confirm observable trends in the presence of random variation. Additionally, the maximum number of generations prior to termination of the simulation, 10,000, was not varied. Nevertheless, I believe that the new approach presented in this article represents a useful tool for exploring various concepts related to evolution in a spatially resolved framework.

## Conclusions and Future Directions

In this study, I demonstrated that the introduction of random mutation into the rule set of the classical GOL algorithm results in the emergence of a highly proliferative, densely packed growing mass of live cells that is not unlike the uncontrolled growth of a cancerous tumor. It was shown that the mutation of the overcrowding rule, to a state more permissive of a neighborhood more densely packed with live cells, is the dominant mechanism in this transition. The simplicity of this model of evolution can be extended in many different directions using the Matlab program shared with the article, for instance to further probe fundamental phenomena in cancer biology. One interesting variation might be to introduce a change in external conditions, such as approximating a drug regimen used to treat a solid tumor or a change in environmental conditions which might cause the “extinction” of subclones of the population. It is well known that drugs such as chemotherapeutics will often successfully kill the majority of tumor cells in an organism, however if a small subpopulation of those cells survive treatment, they can grow back in a drug-resistant form that becomes much harder to treat… a scenario which might be replicated in a GOL-like CA model. One simple improvement to improve the visualization of the simulation would be to color code the cells themselves, such that distinct subclones could be identified in real time as the GOL progresses. Finally, another variation of the GOL + mutation simulation presented here could be to create a scenario approximating “metastasis”. That is, to examine what might happen when a highly mutated cell with increased fitness is transported into a fresh lattice populated solely with wildtype cells… in that case would the mutant “supercell” consistently overgrow and displace the existing cells still carrying the original GOL rule set in its “genetic code”? Perhaps creative scenarios such as this could reveal situations in which one of the other two GOL thresholds, lonely and born, emerge as evolutionary drivers of equal or greater importance to the overcrowding rule.

## Supplementary Information

Below is the link to the electronic supplementary material.

Supplementary file1 (PDF 48 kb)

Supplementary file2 (XLSX 12 kb)

Supplementary file2 (PDF 33 kb)

## Data Availability

Data are provided as a supplemental file.
