# Supplementary material for: Simulation of Somatic Evolution Through the Introduction of Random Mutation to the Rules of Conway’s Game of Life
Source: Cell Mol Bioeng. 2024 Oct 20;17(6):563–71. doi: 10.1007/s12195-024-00828-9 (PMC11799479; doi:10.1007/s12195-024-00828-9)
Supplement: Supplementary file 1 — Supplementary file1 (PDF 48 kb) [file 12195_2024_828_MOESM1_ESM.pdf]

```
function varargout = game_of_life(varargin)
% GAME_OF_LIFE MATLAB code for game_of_life.fig
%      GAME_OF_LIFE, by itself, creates a new GAME_OF_LIFE or raises the existing
%      singleton*.
%
%      H = GAME_OF_LIFE returns the handle to a new GAME_OF_LIFE or the handle to
%      the existing singleton*.
%
%      GAME_OF_LIFE('CALLBACK',hObject,eventData,handles,...) calls the local
%      function named CALLBACK in GAME_OF_LIFE.M with the given input arguments.
%
%      GAME_OF_LIFE('Property','Value',...) creates a new GAME_OF_LIFE or raises the
%      existing singleton*.  Starting from the left, property value pairs are
%      applied to the GUI before game_of_life_OpeningFcn gets called.  An
%      unrecognized property name or invalid value makes property application
%      stop.  All inputs are passed to game_of_life_OpeningFcn via varargin.
%
%      *See GUI Options on GUIDE's Tools menu.  Choose "GUI allows only one
%      instance to run (singleton)".
%
% See also: GUIDE, GUIDATA, GUIHANDLES

% Edit the above text to modify the response to help game_of_life

% Last Modified by GUIDE v2.5 19-May-2015 20:29:16

% Later modified by M.R. King in 2024 for article entitled "Simulation...
% of Somatic Evolution Through the Introduction of Random Mutation to...
% the Rules of Conway's Game of Life

% Begin initialization code - DO NOT EDIT
gui_Singleton = 1;
gui_State = struct('gui_Name',       mfilename, ...
    'gui_Singleton',  gui_Singleton, ...
    'gui_OpeningFcn', @game_of_life_OpeningFcn, ...
    'gui_OutputFcn',  @game_of_life_OutputFcn, ...
    'gui_LayoutFcn',  [] , ...
    'gui_Callback',   []);
if nargin && ischar(varargin{1})
    gui_State.gui_Callback = str2func(varargin{1});
end

if nargout
    [varargout{1:nargout}] = gui_mainfcn(gui_State, varargin{:});
else
    gui_mainfcn(gui_State, varargin{:});
end
% End initialization code - DO NOT EDIT


% --- Executes just before game_of_life is made visible.
function game_of_life_OpeningFcn(hObject, eventdata, handles, varargin)
% This function has no output args, see OutputFcn.
% hObject    handle to figure
% eventdata  reserved - to be defined in a future version of MATLAB
% handles    structure with handles and user data (see GUIDATA)
% varargin   command line arguments to game_of_life (see VARARGIN)

% Choose default command line output for game_of_life
handles.output = hObject;

% Update handles structure
guidata(hObject, handles);
```

```
% UIWAIT makes game_of_life wait for user response (see UIRESUME)
% uiwait(handles.figure1);


% --- Outputs from this function are returned to the command line.
function varargout = game_of_life_OutputFcn(hObject, eventdata, handles)
% varargout  cell array for returning output args (see VARARGOUT);
% hObject    handle to figure
% eventdata  reserved - to be defined in a future version of MATLAB
% handles    structure with handles and user data (see GUIDATA)

% Get default command line output from handles structure
varargout{1} = handles.output;



function edit1_Callback(hObject, eventdata, handles)
% hObject    handle to edit1 (see GCBO)
% eventdata  reserved - to be defined in a future version of MATLAB
% handles    structure with handles and user data (see GUIDATA)

% Hints: get(hObject,'String') returns contents of edit1 as text
%        str2double(get(hObject,'String')) returns contents of edit1 as a double


% --- Executes during object creation, after setting all properties.
function edit1_CreateFcn(hObject, eventdata, handles)
% hObject    handle to edit1 (see GCBO)
% eventdata  reserved - to be defined in a future version of MATLAB
% handles    empty - handles not created until after all CreateFcns called

% Hint: edit controls usually have a white background on Windows.
%       See ISPC and COMPUTER.
if ispc && isequal(get(hObject,'BackgroundColor'),
get(0,'defaultUicontrolBackgroundColor'))
    set(hObject,'BackgroundColor','white');
end



function edit2_Callback(hObject, eventdata, handles)
% hObject    handle to edit2 (see GCBO)
% eventdata  reserved - to be defined in a future version of MATLAB
% handles    structure with handles and user data (see GUIDATA)

% Hints: get(hObject,'String') returns contents of edit2 as text
%        str2double(get(hObject,'String')) returns contents of edit2 as a double


% --- Executes during object creation, after setting all properties.
function edit2_CreateFcn(hObject, eventdata, handles)
% hObject    handle to edit2 (see GCBO)
% eventdata  reserved - to be defined in a future version of MATLAB
% handles    empty - handles not created until after all CreateFcns called

% Hint: edit controls usually have a white background on Windows.
%       See ISPC and COMPUTER.
if ispc && isequal(get(hObject,'BackgroundColor'),
get(0,'defaultUicontrolBackgroundColor'))
    set(hObject,'BackgroundColor','white');
end
```

```
function edit3_Callback(hObject, eventdata, handles)
% hObject    handle to edit3 (see GCBO)
% eventdata  reserved - to be defined in a future version of MATLAB
% handles    structure with handles and user data (see GUIDATA)

% Hints: get(hObject,'String') returns contents of edit3 as text
%        str2double(get(hObject,'String')) returns contents of edit3 as a double


% --- Executes during object creation, after setting all properties.
function edit3_CreateFcn(hObject, eventdata, handles)
% hObject    handle to edit3 (see GCBO)
% eventdata  reserved - to be defined in a future version of MATLAB
% handles    empty - handles not created until after all CreateFcns called

% Hint: edit controls usually have a white background on Windows.
%       See ISPC and COMPUTER.
if ispc && isequal(get(hObject,'BackgroundColor'),
get(0,'defaultUicontrolBackgroundColor'))
    set(hObject,'BackgroundColor','white');
end


% --- Executes on button press in pushbutton1.
function pushbutton1_Callback(hObject, eventdata, handles)
% hObject    handle to pushbutton1 (see GCBO)
% eventdata  reserved - to be defined in a future version of MATLAB
% handles    structure with handles and user data (see GUIDATA)
global filename data

if get(handles.radiobutton1,'Value')==1

    x_length=str2double(get(handles.edit1,'string'));
    y_length=str2double(get(handles.edit2,'string'));

    universe=sign(randn(y_length,x_length));
    %create 3 new arrays that will store the (noninteger) thresholds that
    %occasionally mutate away from Conway's original rules
    lonely=ones(size(universe))*2;
    born=ones(size(universe))*3;
    crowded=ones(size(universe))*3;
    % set the mutation rate and mutation magnitude parameters here
    mutrate=0.1;
    mutmag=0.5;

elseif get(handles.radiobutton2,'Value')==1 & isempty(filename)==0 & filename~=0
    [y_length,x_length]=size(data);
    universe=data;

else
    warndlg('You did not choose a file','The Game of Life')
end
num_gen=str2double(get(handles.edit3,'string'));


universe2=-ones(y_length,x_length);

test_conv=ones(y_length,x_length,3);
test_conv(:,:,1)=universe;
test_conv(:,:,2)=universe2;
```

```
for kk=1:num_gen
    population(kk,1)=sum(universe(:) == 1);
    set(handles.text4,'string',num2str(kk))
    set(handles.text8,'string',num2str(population(kk,1)))

    cla
    pcolor(universe)
    axis off
    axis image
    pause(0.02)

    for i=2:y_length-1
        for j=2:x_length-1
            current_el=universe(i,j);
            small_universe=universe(i-1:i+1,j-1:j+1);
            %need to send the local (mutated) thresholds to f_rules, in
            %integer form
            small_lonely=lonely(i-1:i+1,j-1:j+1);
            small_born=born(i-1:i+1,j-1:j+1);
            small_crowded=crowded(i-1:i+1,j-1:j+1);
            tiny_lonely=round(small_lonely(2,2));
            tiny_born=round(small_born(2,2));
            tiny_crowded=round(small_crowded(2,2));
            % if current_el is dead, must randomly select a neighboring
            % potential "parent" to inherit the born threshold from
            if current_el<0
                indpar=find(small_universe==1);
                if ~isempty(indpar)>0
                    parentind=randi(length(indpar),1);
                    tiny_born=round(small_born(parentind));
                end
            end

universe2(i,j)=f_rules(current_el,small_universe,tiny_lonely,tiny_born,tiny_crowded);
            % find out if a new cell was born. If so, then inherit all
            % thresholds from the parent cell and put in the big matrices
            % also, if a new cell is born, then there is a chance of a
            % random mutation in one of the three thresholds
            if universe2(i,j)>current_el
                lonely(i,j)=small_lonely(parentind);
                born(i,j)=small_born(parentind);
                crowded(i,j)=small_crowded(parentind);
                if rand<mutrate
                    whichthreshold=randi(3,1);
                    if whichthreshold==1
                        lonely(i,j)=lonely(i,j)+randn*mutmag;
                    elseif whichthreshold==2
                        born(i,j)=born(i,j)+randn*mutmag;
                    else
                        crowded(i,j)=crowded(i,j)+randn*mutmag;
                    end
                end
            end
            % if the current cell just died, then reset all thresholds in
            % the big matrices back to their Conway defaults
            if universe2(i,j)<current_el
                lonely(i,j)=2;
                born(i,j)=3;
                crowded(i,j)=3;
            end
        end
    end
```

```
    universe=universe2;
    test_conv(:,:,3)=test_conv(:,:,2);
    test_conv(:,:,2)=test_conv(:,:,1);
    test_conv(:,:,1)=universe;

    test1=test_conv(:,:,3)-test_conv(:,:,1);

    if max(max(test1))==min(min(test1))
        answer = questdlg('The cell population has converged. Would you like to plot 
the population?','Game of Life','Yes','No','Yes');
        if strcmp(answer,'Yes')==1
            figure
            plot(population)
            title('Population','Fontsize',15,'Fontweight','bold')
            xlabel('Number of cells','Fontsize',15,'Fontweight','bold')
            ylabel('Generation','Fontsize',15,'Fontweight','bold')
            set(gca,'Fontsize',15)
            % now plot up the chessboards of the mutated thresholds
            figure
            pcolor(lonely)
            title('final loneliness thresholds')
            figure
            pcolor(born)
            title('final born thresholds')
            figure
            pcolor(crowded)
            title('final crowded thresholds')
            meanlonely=mean(mean(lonely))
            meanborn=mean(mean(born))
            meancrowded=mean(mean(crowded))
        end
        return
    end

end

answer = questdlg('Generations have been completed. Would you like to plot the 
population?','Game of Life','Yes','No','Yes');
if strcmp(answer,'Yes')==1
    figure
    plot(population)
    title('Population','Fontsize',15,'Fontweight','bold')
    xlabel('Number of cells','Fontsize',15,'Fontweight','bold')
    ylabel('Generation','Fontsize',15,'Fontweight','bold')
    set(gca,'Fontsize',15)
    % now plot up the chessboards of the mutated thresholds
    figure
    pcolor(lonely)
    title('final loneliness thresholds')
    figure
    pcolor(born)
    title('final born thresholds')
    figure
    pcolor(crowded)
    title('final crowded thresholds')
    meanlonely=mean(mean(lonely))
    meanborn=mean(mean(born))
    meancrowded=mean(mean(crowded))

end

% --- Executes on button press in radiobutton1.
function radiobutton1_Callback(hObject, eventdata, handles)
```

```
% hObject    handle to radiobutton1 (see GCBO)
% eventdata  reserved - to be defined in a future version of MATLAB
% handles    structure with handles and user data (see GUIDATA)

% Hint: get(hObject,'Value') returns toggle state of radiobutton1
set(handles.radiobutton1,'Value',1)
set(handles.edit1,'Enable','on')
set(handles.edit2,'Enable','on')

set(handles.radiobutton2,'Value',0)
set(handles.pushbutton2,'Enable','off')

% --- Executes on button press in radiobutton2.
function radiobutton2_Callback(hObject, eventdata, handles)
% hObject    handle to radiobutton2 (see GCBO)
% eventdata  reserved - to be defined in a future version of MATLAB
% handles    structure with handles and user data (see GUIDATA)

% Hint: get(hObject,'Value') returns toggle state of radiobutton2
set(handles.radiobutton1,'Value',0)
set(handles.edit1,'Enable','off')
set(handles.edit2,'Enable','off')

set(handles.radiobutton2,'Value',1)
set(handles.pushbutton2,'Enable','on')

% --- Executes on button press in pushbutton2.
function pushbutton2_Callback(hObject, eventdata, handles)
% hObject    handle to pushbutton2 (see GCBO)
% eventdata  reserved - to be defined in a future version of MATLAB
% handles    structure with handles and user data (see GUIDATA)
global filename data

filename=[];
[filename, pathname] = uigetfile('*.*','File Selector');

if (filename~=0)
    datafile= fullfile(pathname,filename);
    data=importdata(datafile);
end


function [output] =
f_rules(current_el,small_universe,tiny_lonely,tiny_born,tiny_crowded)

if current_el==1 %The current cell is alive
    if sum(small_universe(:) == 1)<(tiny_lonely+1) %The current cell has fewer than 2
live neighbours
        output=-1; %The current cell dies by under-population
    elseif sum(small_universe(:) == 1)>tiny_lonely && sum(small_universe(:) ==
1)<(tiny_crowded+2) %The current cell has 2 or 3 live neighbours
        output=1; %The current cell lives on to the next generation
    elseif sum(small_universe(:) == 1)>(tiny_crowded+1)
        output=-1; %The current cell dies by over-population
    end
elseif current_el==-1 %The current cell is dead
    if sum(small_universe(:) == 1)==tiny_born %The current cell has exactly 3 live
neighbours
        output=1; %The current cell becomes alive
    else
        output=-1; %The current cell stays dead
    end
end
```
