# Supplementary material for: Simulation of Somatic Evolution Through the Introduction of Random Mutation to the Rules of Conway’s Game of Life
Source: Cell Mol Bioeng. 2024 Oct 20;17(6):563–71. doi: 10.1007/s12195-024-00828-9 (PMC11799479; doi:10.1007/s12195-024-00828-9)
Supplement: Supplementary file 3 — Supplementary file2 (PDF 33 kb) [file 12195_2024_828_MOESM3_ESM.pdf]

List of Supplementary Videos:

Supplementary Video 1: Animation of simulation run in the absence of mutation (mutation rate = 0).

Supplementary Video 2: Animation of simulation run with mutation rate = 0.01 and mutation magnitude = 1.0.

Supplementary Video 3: Animation of simulation run with mutation rate = 0.05 and mutation magnitude = 6.0.

Supplementary Video 4: Animation of simulation run with a mutation rate = 0.01 and a mutation magnitude = 5.0.

Supplementary Video 5: Animation of simulation run with a mutation rate = 0.05 and a mutation magnitude = 0.5.

Supplementary Video 6: Animation of simulation run with a mutation rate = 0.05 and a mutation magnitude = 1.0.

Supplementary Video 7: Animation of simulation run with a mutation rate =0.4 and a mutation magnitude = 1.5.

Supplementary Video 8: Animation of simulation run with a mutation rate =0.1 and a mutation magnitude = 0.5.

Note: All supplementary videos are publicly available at: https://zenodo.org/records/12575091
